# Hyperthermic Overdrive: Oxygen Delivery does Not Limit Thermal Tolerance in *Drosophila melanogaster*

**DOI:** 10.1673/031.012.10901

**Published:** 2012-09-07

**Authors:** Andreas B. Mölich, Thomas D. Förster, John R. B. Lighton

**Affiliations:** ^1^ Sable Systems Europe, Sable Systems Europe GmbH, Ostendstr. 25, 12459 Berlin, Germany; ^2^ Sable Systems International, 6000 S. Eastern Ave., Las Vegas, NV 89119, USA; ^3^ Department of Biological Sciences, University of Nevada at Las Vegas, 4505 Maryland Parkway, NV 89154, USA

## Abstract

The causes of thermal tolerance limits in animals are controversial. In many aquatic species, it is thought that the inability to deliver sufficient oxygen at high temperatures is more critical than impairment of molecular functions of the mitochondria. However, terrestrial insects utilize a tracheal system, and the concept of a mismatch between metabolic demand and circulatory performance might not apply to them. Using thermo-limit respirometry, it has been shown earlier in *Drosophila melanogaster* that CO_2_ release rates at temperatures above the upper thermal limit (CT_max_) exceed the rate at CT_max_. The nature of this post-CT_max_, or “post-mortal” peak, is unknown. Either its source is increased aerobic mitochondrial respiration (hyperthermic overdrive), or an anaerobic process such as liberation of stored CO2 from the hemolymph. The post-mortal peak of CO2 release was found to be oxygen dependent. As the rate of CO_2_ emission is a conservative indicator of rate of O_2_ consumption, aerobic flux at the thermal limit is submaximal, which contradicts the theory that oxygen availability limits metabolic activity at high temperatures in insects. Consequently, the tracheal system should be capable of delivering sufficient oxygen for aerobic activity of the mitochondria at and above Ct_max_.

## Introduction

Maximal oxygen supply in various animals is restricted either by the physical properties of the environment in which they live, or by the need to balance the components of the respiratory cascade (Weibel et al. 1992), and often by both factors simultaneously. Aquatic species are affected by the low solubility and slow diffusion of O2 in water and body fluids as compared to air, approximately 30 and 105 times lower, respectively (Dejours 1975). Species relying on convective oxygen supply must modulate ventilation, circulation, and tissue perfusion in accord with mitochondrial activity.

Though ectotherms evolved to meet high metabolic demands during phases of aerobic activity, their respiratory and circulatory systems are substantially challenged by thermal stress. At high temperatures, oxygen delivery to the mitochondria may become insufficient to sustain aerobic metabolism. This is because the relationship between temperature and metabolic activity is exponential, at least until thermal stress, through whatever mechanism, causes a downward inflection in metabolic activity as CT_max_ is approached. The concept of oxygen-limited and capacity-limited thermal tolerance states that the capacities of oxygen supply systems at the whole-organism level, rather than temperature responses at the cellular or molecular level, confine the thermal windows within which metazoans operate (Pörtner 2001). Originally developed for marine invertebrates and fishes (Frederich and Pörtner 2000, Pörtner et al. 2004), this hypothesis may be more generally applicable to a number of air and water breathing species (Pörtner 2001).

However, insects circumvent the above mentioned respiratory restrictions for liquid phase oxygen transport. Their respiratory system, the tracheal system, consists of a finely branched, gas-filled tubular network, which directly connects the outside air with the target tissues. Given the relatively small size of insects, the tracheal system is highly effective. Except for special cell types (Locke 1997), hemolymph circulation is generally considered unnecessary for adequate oxygen supply. The hemolymph contains only a few O_2_-binding pigments, the function of which is unclear (Burmester and Hankeln 1999). At rest, ventilation often is discontinued, and diffusion in the tracheal system alone can supply adequate oxygen (Lighton 1996, Wobschall and Hetz 2004). Overall, the oxygen supply chain is highly simplified to a single transport system in insects. According to the theory of capacity-limited thermal tolerance, the performance of this system alone would then define the thermal limits of the animal.

**Figure 1.**
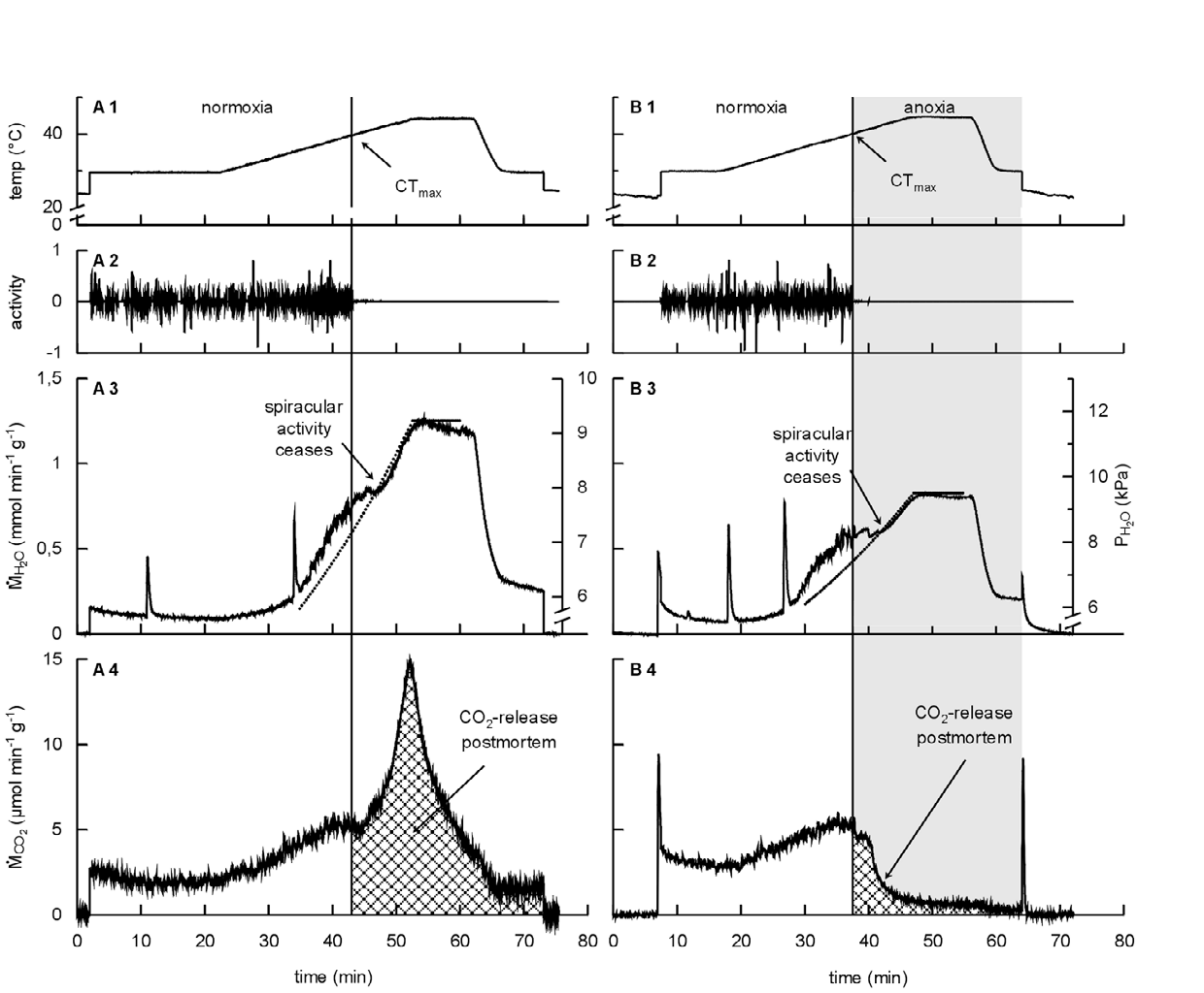
Typical effect of anoxia on the metabolic response to thermal stress in *Drosophila melanogaster*. Panel A shows the response in normoxia. Panel B shows the effect of switching from normoxic to anoxic conditions immediately after the maximum critical temperature (CT_max_) of the fly was reached (shaded area in panel B). 1: The temperature ramp. 2: Activity (determined photoelectrically; arbitrary units). 3: Water loss rate. The sharp upward deflections in the water loss traces before CT_max_ are excretion events. The dotted curves show the saturated water vapor pressure at the temperature of the water vapor trace (to which passive water loss rate is proportional). 4: CO_2_ emission rate. The extirpation of the post-mortal CO_2_ peak in anoxia is clearly shown (cross-hatched areas). High quality figures are available online.

It has been suggested that capacity limitation of thermal tolerance is not significant in at least some tracheate arthropods (Klok et al. 2004), using the new technique of thermolimit respirometry (Lighton and Turner 2004). Thermo-limit respirometry allows the simultaneous measurement of the upper thermal limit and carbon dioxide emission. A peculiar feature of this method in insects is a post-mortal CO_2_ peak (Figure 1a) which occurs after the death of the animal. However, CO_2_ release is not a reliable indicator of metabolic demand/oxygen consumption. Lowering of tissue or hemolymph pH, resulting in liberation of CO_2_ from bicarbonate, as well as other anaerobic processes, can significantly distort the relationship between oxygen consumption and carbon dioxide release. Thus, the origin of the peak has remained unclear. If the oxygen capacity limitation hypothesis holds, the peak must be of anaerobic origin (see discussion for details). Therefore, elucidating the nature of the mechanism behind it provides a further direct test of the capacity limitation hypothesis.

## Materials and Methods

### Animals

*Drosophila melanogaster*, strain Oregon-R wild type, were reared at 25° C with a 12: 12 L:D cycle. Adult flies were released, and flies eclosing over the subsequent two days were collected in narrow vials containing feeding medium. Flies aged 5–10 days (approximately equally divided between male and female) were used for the experiments described here.

### Thermolimit respirometry

To determine whether the high post-CT_max_ CO_2_ release rate is coupled to aerobic processes, CO_2_ flux, evaporative water loss, and behavioral activity of individual *D. melanogaster* were monitored using a Sable Systems SI-1 respirometry system (Sable Systems International, Las Vegas, Nevada, USA). To determine thermal limits, after an equilibration period of ∼10–20 min at 30° C the temperature of the fly was continuously increased from 30 to 45° C at a rate of 0.5° C min-1. Refer to Lighton (2007) for a detailed methodological description of the setup. The thermo-limit respirometry method is described in detail by Lighton and Turner (2004), including the requisite data analysis and statistical methods.

**Table 1.**
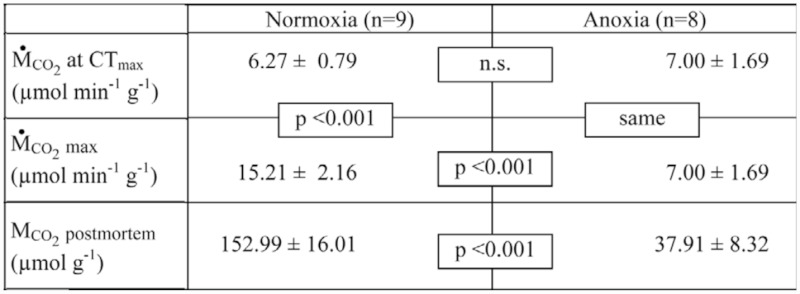
Carbon dioxide release rates during thermo-limit respirometry in normoxia and anoxia in *Drosophila melanogaster*.

In the absence of oxygen, all aerobic processes quickly cease, without directly affecting anaerobic processes. Thus, anoxia treatment was used to infer the nature of the post-mortal peak. Chamber air was immediately switched to nitrogen using a computer-controlled solenoid valve as soon as the activity of the flies ceased, and the carbon dioxide and water traces signified that the upper thermal limit had been reached. Control experiments were conducted with normoxia maintained throughout the whole trial.

## Results

Figure 1, Panel A, shows a typical thermolimit respirometry experiment in normoxia. After an initial recovery phase of 20 minutes, temperature slowly increased from 30° C to 45° C. Death occurred at CT_max_ = 39.6 ± 1.0° C (n = 17), when all body movements ceased. Below CT_max_, CO_2_ release rate entered a plateau, and evaporative water loss rate increased more slowly than at temperatures above CT_max_, indicating that neuromuscular control of the spiracles was still intact (Lighton and Turner 2004; Klok et al. 2004; Lighton 2007). These short-term variations in CO_2_ and H_2_O flux then ceased abruptly above CT_max_, and were followed in normoxia by the post-mortal CO_2_ peak. Carbon dioxide release rate at CT_max_ was 6.27 µmol g^-1^ min^-1^, and maximal post-CT_max_ rate was 15.21 µmol g^-1^ min^-1^ (Table 1).

Anoxia resulted in exponential decay of CO_2_ emission rate, and total extirpation of the postmortal CO_2_ peak (Figure 1, panel B). At CT_max_, the CO_2_ release of both groups did not differ significantly, but the maximal postmortem CO_2_ release rate in the anoxic group was significantly lower (p < 0.001, Table 1). In normoxia, maximal CO_2_ release rate exceeded the CT_max_ rate by a factor of two or more (p < 0.001), while in anoxia, maximal CO_2_ release occurred at CT_max_ just prior to exposure to anoxia. In addition, the total amount of CO_2_ released in the 20 minute period following CT_max_ was four-fold lower in the anoxic treatment (p < 0.001). Note that anoxia does not reduce the degree of spiracular opening; instead, it maximizes it (Lighton and Schilman 2007), as can also be seen from the water vapor trace (Figure 1). So, the observed reduction in the post-mortal peak is not caused by spiracular closure.

With respect to evaporative water loss, we compared the ratio of water loss rate before and after CT_max_ in the control and anoxia-exposed groups, which were 0.268 ± 0.012 SD and 0.284 ± 0.026 SD, respectively. These ratios did not differ significantly (after arcsine-transforming the square root of the ratios to normalize them, t = 0.52, df = 15, *p* = 0.6). After the initial increase of water loss driven by the increasing water vapor saturation deficit, increase in evaporation rate slowed down, probably due to increased spiracular control near CT_max_. After spiracular control ceased above CT_max_, water loss rate increased with a rate comparable to the initial saturation deficit driven increase, as can clearly be seen in Figure 1, panels A3 and B3, indicating constant and likely maximal spiracular aperture post-mortem.

## Discussion

The results clearly demonstrated an aerobic origin of the post-mortal peak. Thus, the null hypothesis that the peak results from anaerobic washout of internal CO2 stores is rejected. Another alternative explanation, that a change of tracheal conductance is due to post mortem increase in spiracular aperture, is inconsistent with the observed rates of evaporative water loss.

In insects, water loss occurs either via the external body surface, or via the spiracles located at the distal opening of the tracheal tubes. For a given gradient of water vapor pressure between body and environment, surface evaporation is constant, since the body surface area does not change. Respiratory water loss is determined by the aperture of the spiracular valves, which are under CNS control. The effect of this spiracular control is seen as small, noise-like variations in the water vapor trace in Figure 1, panels A3 and B3. At CT_max_, this flicker in the water vapor trace ceases, indicating loss of spiracular control and constant spiracular aperture. Throughout the experiment, water loss rates approximately track the water vapor pressure saturation deficit of the air within the chamber; this is clearly seen in Figure 1, panels A3 and B3.

The CO_2_ release rate of approximately 7 µmol g^-1^ min^-1^ at CT_max_ in *D. melanogaster*, determined in this study and previous studies (Lighton 2007), is far lower than the values of approximately 30 µmol g^-1^ min^-1^ found for *D. melanogaster* flying at maximal performance (Lehmann 2001). The peak post-mortem rate of CO_2_ production, while more than twice the rate recorded at CT_max_, is still only half the maximum rate recorded during flight at room temperature. A possible explanation may be that in flies, after death, oxygen supply is purely diffusive, whereas convection and autoventilation could play a dominant role during flight (Komai 1998).

However, this does not affect the conclusions derived from our results. Since the post-mortal peak is aerobic, the increased rate of carbon dioxide release past CT_max_ indicates that the tracheal system is capable of delivering more oxygen than is required at CT_max_. While oxygen consumption was not measured directly, the rate of CO_2_ emission is a conservative indicator of the rate of O_2_ consumption. The constant of diffusion for O_2_ in air is only slightly larger than that of CO_2_, but the difference in partial pressure that drives diffusion between outside air and the mitochondria is approximately four-fold greater for O_2_. The maximal mismatch between O_2_ consumption and CO_2_ production during normal aerobic activity does not exceed 1:0. 72 (respiratory quotient of 0.72). Thus, in steady state conditions, CO_2_ excretion is more likely to be a limiting factor than O_2_ supply. Often, CO_2_ release rate more than doubled post mortem in normoxia, evidencing a substantial increase of oxygen consumption after the animal died.

Clearly, contrary to what the oxygen limitation hypothesis predicts, in insects the upper thermal limit is not defined by the oxygen supply capacity of the tracheal system. The results of this study, therefore, support the conclusions of Klok et al. (2004), and provide further evidence that the oxygen limitation hypothesis does not apply to tracheate arthropods, at least in normoxia. The mitochondrial mechanism behind the postmortal CO2 peak was not investigated. While of great theoretical interest, it does not affect the conclusions derived above. Mitochondrial “hyperthermic overdrive” may originate from thermally mediated membrane breakdown, and the resulting uncoupling of oxidative phosphorylation. Other workers are encouraged to explore this interesting phenomenon in greater detail.

## References

[bibr01] Burmeister T, Hankeln T (1999). A Globin Gene of *Drosophila melanogaster*.. *Molecular Biology and Evolution*.

[bibr02] Dejours P (1975). *Principles of Comparative Respiratory Physiology*..

[bibr03] Frederich M, Pörtner HO (2000). Oxygen limitation of thermal tolerance defined by cardiac and ventilatory performance in spider crab, *Maja squinado*.. *American Journal of Physiology*.

[bibr04] Klok CJ, Sinclair BJ, Chown SL (2004). Upper thermal tolerance and oxygen limitation in
terrestrial arthropods.. *Journal of Experimental Biology*.

[bibr05] Komai Y (1998). Augmented respiration in a flying insect.. *Journal of Experimental Biology*.

[bibr06] Lehmann F (2001). Matching Spiracle Opening to Metabolic Need During Flight in *Drosophila*.. *Science*.

[bibr07] Lighton JRB (1996). Discontinuous Gas Exchange in Insects.. *Annual Review of Entomology*.

[bibr08] Lighton JRB (2007). Hot hypoxic flies: Whole-organism interactions between hypoxic and thermal stressors in *Drosophila melanogaster*.. *Journal of Thermal Biology*.

[bibr09] Lighton JRB, Schilman PE (2007). Oxygen reperfusion damage in an insect.. PLoS ONE.

[bibr10] Lighton JRB, Turner RJ (2004). Thermolimit respirometry: an objective assessment of critical thermal maxima in two sympatric desert harvester ants, *Pogonomyrmex rugosus* and *P. californicus*.. *Journal of Experimental Biology*.

[bibr11] Locke M (1997). Caterpillars have evolved lungs for hemocyte gas exchange.. *Journal of Insect Physiology*.

[bibr12] Pörtner HO (2001). Climate change and temperature-dependent biogeography: oxygen limitation of thermal tolerance in animals.. *Naturwissenschaften*.

[bibr13] Pörtner HO, Mark FC, Bock C (2004). Oxygen limited thermal tolerance in fish? Answers obtained by Nuclear Magnetic Resonance techniques.. *Respiratory Physiology & Neurobiology*.

[bibr14] Weibel ER, Taylor CR, Hoppeler H (1992). Variations in function and design: Testing symmorphosis in the respiratory system.. *Respiratory Physiology & Neurobiology*.

[bibr15] Wobschall A, Hetz SK (2004). Oxygen uptake by convection and diffusion in diapausing moth pupae (*Attacus atlas*).. *International Congress Series*.

